# The Potential Role of Insects as Feed: A Multi-Perspective Review

**DOI:** 10.3390/ani9040119

**Published:** 2019-03-27

**Authors:** Giovanni Sogari, Mario Amato, Ilaria Biasato, Silvana Chiesa, Laura Gasco

**Affiliations:** 1 Department of Food and Drug, University of Parma, 43124 Parma, Italy; silvana.chiesa@gmail.com; 2 Charles H. Dyson School of Applied Economics and Management, Cornell University, Ithaca, NY 14850, USA; 3 Department of Political Sciences, University of Naples Federico II, 80138 Naples, Italy; mario.amato2@unina.it; 4 Department of Agricultural, Forest, and Food Sciences, University of Turin, Grugliasco, 10124 Torino, Italy; ilaria.biasato@unito.it (I.B.); laura.gasco@unito.it (L.G.)

**Keywords:** consumer, stakeholder, regulations, sustainability, animals, farming, acceptance, protein, feed meal, novel

## Abstract

**Simple Summary:**

The consumption of meat and fish is growing, and it is becoming increasingly difficult to meet the growing demand for protein by farming on land and in water, causing serious repercussions for the planet’s resources, socio-economic development, and environmental sustainability. The search for new food solutions with good nutritional value for direct and indirect human consumption is of fundamental importance. The use of insects for feeding farmed animals represents a promising alternative because of the nutritional properties of insects and the possible environmental benefits, given the sustainability of this type of farming. Yet, there is a lack of consensus among western consumers. Therefore, the aim of this paper is to report and discuss previous consumer and stakeholder studies related to insects as feed, including the main market challenges for this novel source. The results show that, despite a sparse body of research, consumer acceptance will not be a barrier towards the development of the insect protein industry for feed. However, further research should shed light on consumer willingness to pay for animal products from animals fed with insects and whether the overall acceptability, in general and from a sensory point of view, will be perceived better than conventional products.

**Abstract:**

Recently, insects have received increased attention as an important source of sustainable raw materials for animal feed, especially in fish, poultry, and swine. In particular, the most promising species are represented by the black soldier fly (*Hermetia illucens*, HI), the yellow mealworm (*Tenebrio molitor*, TM), and the common house fly (*Musca domestica*, MD). Although rapid development is expected, insects remain underutilized in the animal feed industry mainly due to technical, financial, and regulatory barriers. In addition, few works have analyzed consumer and stakeholder points of view towards the use of insects as animal feed. In this article, we summarize the main findings of this body of research and provide a discussion of consumer studies regarding the consumption of animals fed with insects. Our review suggests that consumer acceptance will not be a barrier towards the development of this novel protein industry. Furthermore, we conclude that it will be of interest to understand whether the use of this more sustainable feed source might increase consumer willingness to pay for animal products fed with insects and whether the overall acceptability, from a sensory point of view, will be perceived better than conventional products. Finally, the main challenges of the feed farming industry are addressed.

## 1. Introduction

The major global shift towards diets characterized by the increased consumption of animal products and the growing demand for feed ingredients is likely to continue in the near future, and the quest for alternative sustainable animal protein sources is expected to become a considerable issue in the feed market [1,2,3]. Over the last few years, insects have been identified as an important future source of sustainable raw materials for animal feeds in many countries around the world. First, insects meet animals’ dietary requirements in terms of nutritional composition, amino acid profile, and, as part of the natural diet of several animal species, feed acceptance [4]. Mass production of insects is also promising from an environmental perspective because of the low emission levels of greenhouse gases [5], the small land area needed to produce 1 kg of protein [6], the reduction of land area utilization as a consequence of the lower feed–food competition [4], and the ability to convert organic side streams into high-value protein products [7]. In particular, the use of insects in the bioconversion of waste materials constitutes a novel approach and a remarkable example of a sustainable circular economy [7]. Several insects have been tested as animal feeds, with the most promising species being the black soldier fly (*Hermetia illucens*, HI) [8,9], the yellow mealworm (*Tenebrio molitor*, TM) [10,11], and the common house fly (*Musca domestica*, MD) [12,13]. Their high potential as alternative feed ingredients is related to the possibility of controlling their life cycle process and, thus, mass rearing them [14], as well as considerations of competitive trading prices among the species proposed as animal feeds [15]. Previous research has highlighted the possibility of including insect larva/prepupa meal/fat in fish [1,3,16], poultry [17,18,19], and weaning pig [19,20,21] diets as partial or total replacement of conventional protein/fat sources (soybean and fish meals and oils), which are no longer considered sustainable [4]. Positive results have been observed in terms of animal health and performance, gut health aspects, and product quality. The utilization of insects as novel feed additives to improve gut health has also attracted increasing interest, because they contain bioactive components, such as lauric acid, antimicrobial peptides, and chitin, which have immune-boosting properties [22,23]. Within the market scenario, the insect business is growing fast. Since 2000, several companies have been founded in the United States of America (USA), Canada, China, South Africa, and Europe. The overall growth of the insect-rearing sector is particularly related to the growth of the HI-producing companies. Indeed, the global production of HI has grown rapidly, moving from 7000–8000 tons wet weight in 2014–2015 to 14,000 tons in 2016 [15]. This positive market trend may reflect the benefits that stakeholders obtain from producing insects, which, in turn, potentially derive from meeting the consumers’ awareness of the negative impact of animal-derived food production on the environment. Other benefits include the reduction of the disposal costs of organic side streams and valorizing value-added animal products in the poultry meat industry.

Because insects are consumed naturally by many animals, including fish, wild birds, and free-range poultry, we can assume that these animals are evolutionarily adapted to eating them as a part of their regular diet [24,25]. Therefore, it seems reasonable to consider insect proteins as a plausible commercial feed source in the coming years. However, there is less research concerning the point of view of consumers and stakeholders towards the use of insects as livestock feed.

The main aim of this study is to report and discuss previous consumer and stakeholder studies related to insects as feed, including the main market challenges for this novel source. The article is organized as follows: After this introduction, Section 2 provides an outlook of the legislative framework to introduce this new alternative protein source in the animal farming industry. Section 3 reviews and discusses the findings of studies on consumers’ and stakeholders’ preferences towards insects as feed, as well as sensory tests on animals fed with different insect meals. The last section concludes the paper and provides perspectives on the current challenges and future research in this sector.

## 2. Insects as Feed: The Legal Framework

The regulatory system on the use of insects as feed differs widely between countries worldwide [26,27,28,29,30,31] and is not always related to the “traditional” use of insects as food. The following section presents a concise review of the main elements of the existing legislation on the use of insects as feed in the European Union, North America (the United States of America and Canada), and some Asian countries (China, North Korea, and South Korea). The main points are reported in Table 1.

### 2.1. European Union

The European approach to insects as feed is greatly affected by the issue of Bovine Spongiform Encephalopathy (BSE), which poses a serious threat to consumer health and safety [28,32]. In 2001, the use of processed animal proteins (PAPs) was banned owing to transmissible BSE regulation [33]. The next year, Directive 2002/32 [34] established the materials intended for use in animal feed, as well as undesirable substances and their maximum allowable levels in feed (a complete list in Annex I of Directive 2002/32). This is important, because every successive element included in feed (whole insects or insect ingredients) must comply with the limits on undesirable substances. After a few years (thanks to the good management of BSE control practices), the PAPs prohibition was amended and Regulation 56/2013 [35] led to the use of PAPs from any source, except ruminants, in aquaculture. Nevertheless, insects were not yet specifically regulated in the raw material catalogue (Regulation (UE) 68/2013).

Regulation (EU) 2017/893 [36], which amends Annexes I and IV to Regulation (EC) No. 999/2001 and Annexes X, XIV, and XV to Commission Regulation (EU) No. 142/2011, regards the provisions on processed animal protein. Regulation (EU) 2017/893 authorizes the feeding of non-ruminant processed animal protein to aquaculture animals only. Annex II lists processed animal protein derived from farmed insects intended for the production of feed for farmed animals other than fur animals. So far, only seven species are permitted for use: the house cricket (*Acheta domesticus*), banded cricket (*Gryllodes sigillatus*), field cricket (*Gryllus assimilis*), yellow mealworm (*Tenebrio molitor*), lesser mealworm (*Alphitobius diaperinus*), black soldier fly (*Hermetia illucens*), and the common house fly (*Musca domestica*); the regulation also specifies the substrates allowed as feed for insects.

In 2017, Regulation (EU) 2017/1017 [37] amended Regulation (EU) No. 68/2013 [38] with respect to the catalogue of feed materials and permitted the use of live terrestrial invertebrates and dead terrestrial invertebrates with or without treatment as feed materials, but not as processed as described in Regulation (EC) No. 1069/2009. Thereby, terrestrial invertebrates are considered appropriate materials for feed in all their life stages, with the exception of species having adverse effects on plant, animal, or human health. Today, the use of fat from insects is permitted to be used in feeding every animal species, but PAPs are only permitted for use in aquaculture. However, the possibility of extending the authorization of their use to poultry and swine feed is under discussion.

### 2.2. North America

The authority responsible for controlling the safety of animal feed in the USA, is the Federal Food and Drug Administration (FDA), which collaborates with the Association of American Feed Control Officers (AAFCO) in the area of feed regulation, particularly in addressing new feed ingredients [27]. AAFCO is composed of state, federal, and international regulatory officials who are responsible for the enforcement of state laws regulating the safe production and labeling of animal feed.

Edible insects are considered to be food additives in the United States [27], and the annual AAFCO Official Publication, which contains the most complete list of feed ingredients with their definitions, includes the list of approved food additives, as well as the list of generally recognized as safe (GRAS) substances. Today, only HI has been included as an ingredient for animal feed in the both forms (dried whole larvae and HI meal), and its use is limited to aquaculture (i.e., salmonid fish).

In Canada, the Animal Feed Division, Animal Health Directorate, of the Canadian Food Inspection Agency (CFIA) is the authority in charge of managing the Food Act and Feeds Regulation of 1983; it also registers feed and feed ingredients and develops feed-related policies [26].

In Canada, insects are considered to be novel feeds, which are those ingredients that do not have a history of safe use. In accordance with the Food Act, novel feeds and their process of registration are included in Paragraph 4 (Deleterious Substance), Subparagraph 4.1 (Notification and Authorization of the Release of Novel Feeds), in which the focus is on risk assessment for animal health and the environment. Each registration proposal must detail the insect species, their specific rearing condition, and the substrate on which the insects were grown and fed. In 2016, the use of HI larvae was authorized for chicken feed, and in 2017, it was authorized for use in aquaculture. In 2018, the authorization was extended to all poultry.

### 2.3. Asia

In several Asian countries, insects have been historically considered food and feed and used as a good source of protein. In China, there are no specific laws for their regulation. Insects can also be used as feed additives, and in this case, producers must respect the rules collected in the Administrative Measures for Feed and Feed Additives [26].

Another example is the differing approaches of North Korea (Democratic Republic of Korea) and South Korea (Republic of Korea). These two countries, despite having the same history (almost until the twentieth century), language, culture, and food culture, currently have a completely different approach to insects as food and feed. According to Jo and Lee [39] in North Korea, there are legal problems that affect the use of insects as feed, because insects are considered to be animal-based protein, and these are banned for use in animal feed. On the other hand, in South Korea, insects are considered to be a historical component of the human diet and are included in animal feed [31]. There are no specific rules about insects as food and feed because of the deregulation of legislation concerning insects decided by the South Korean government in 2015 [31].

## 3. Are Consumers Ready for Insects as Feed?

In most European countries so far, insects have been generally used for pet feed (such as birds, reptiles, and amphibians) rather than for human or farm animal consumption. However, this trend seems to be changing mainly due to research efforts in this field [30,40,41].

In a global context, where the food supply cannot keep up with the continuous demographic and urban development, institutions and academics have rigorously been looking for alternative sources of protein for human consumption. As was briefly mentioned in the introduction, the farming and processing of insects for human consumption and the production of animal feed has a number of advantages for the environment, health, the improvement of the social condition, and the means of subsistence of various populations [42].

Recently, the scientific literature has mainly focused on the study of westerners’ acceptability of insects as food, highlighting the role played by neophobia [43], disgust [44,45,46], familiarity (that is, a previous instance of consumption), and the distinction between processed and unprocessed insects [47,48,49]. Furthermore, a plethora of studies have showed that consumers are more prone to trying processed and less-visible insects [50,51].

Therefore, a way to augment entomophagy acceptance might be the use of insects as feed instead of as “raw” or “processed”. Yet, despite consumers’ perception, patterns of consumption and willingness to try insects as food have been well documented, although little is known about consumers’ opinions and attitudes towards insects used as feed.

We searched for publications and resources among relevant materials that were already familiar. Then, after a first screening of keywords that best matched our research question (“what is known about animal fed containing insects from a consumer perspective?”), an electronic search in several academic databases was executed. The main exclusion criteria for this review was to omit non-primary research studies (i.e., reviews, commentaries, and editorials).

Only six relevant works were identified based on a literature search conducted in Web of Knowledge, Scopus, and Business Source Complete using combinations of the following keywords: consum* AND insect* AND feed in TITLE-ABSTRACT-KEYWORDS. The search was restricted to English-language, published articles. No date restrictions were applied. We also identified other relevant references (i.e., through gray literature) that met the inclusion criteria but were not initially found in the online database search. We present a total of eight studies about consumers’ acceptance and awareness of insects as feed in Table 2; we provide a summary of each, which includes the study’s method, country, sample, information of what animal was fed with insects, and main findings.

### 3.1. Consumer Acceptance

In a first exploratory attempt, the PROteINSECT European project [52] carried out two surveys between 2013 and 2014 and in 2015. The surveys showed a general consensus around the topic. In particular, even though 64% of participants rated the health risk of eating farmed animals fed with insects as “no” or “low risk”, 88% of the sample asked for more information, pointing out a lack of knowledge regarding the subject. Verbeke et al. [53] surveyed Belgian farmers, agricultural sector stakeholders, and citizens. Despite the fact that the sample was not representative of the overall study population, their findings showed that interviewees’ attitudes and reception were generally in favor of the utilization of insects as animal feed, in particular for fish and poultry. In 2016, two surveys were carried out [54,55] addressing Italian respondents’ perceptions of the use of insects as an alternative meal for fish and livestock. Laureati and colleagues [54] interviewed 341 students and employees of the University of Milan and consumers unrelated with academia in order to compare different perspectives on the subject. In the first step, bystanders completed a survey, while a subset of the same sample was also involved in a second experiment, in which they were asked to evaluate visual appearances of insect-based food products. Following the results of the survey, in which insects as feed were investigated, 53% of the sample reported that they were ready to accept the incorporation of insects into animal diets (as supplements) and to eat animals reared this way. The authors then categorized consumers into three different groups: the “willing”, the “uncertain”, and the “unwilling”, showing how the percentages of these three categories of possible future consumers vary when indirect consumption of insects is assumed (feed) compared with direct consumption (food).

Similarly, Mancuso et al. [55] carried out a face-to-face interview of 277 Northern Italian fish consumers. The authors pointed out that, despite a broad consensus around the topic, the positive attitude shown by a larger percentage of their sample was mainly driven by interest in the topic, whereas the willingness to buy fish fed with insects is strictly connected to selling price, as long as hygiene requirements are met. On the other hand, consumers who were unwilling to consume fish fed with insects based their rejection on the possible “distaste” and a lack of trust in the production process.

Other surveys have been carried out in Poland [58] and the United Kingdom (UK) [59]. In a prior study, the sample mainly favored insects as feed for birds (58.1% of the sample) and fish (56.7%) compared with cattle and pigs, where a lack of willingness to consume was noticed. The authors argued that this discrepancy can be derived from the fact that insects can be part of bird and fish diets, reared both in natural conditions or in various breeding systems (e.g., free-range farming for poultry). Popoff and colleagues [59] investigated, instead, the incorporation of cultured insect larvae into commercial formulated fish feeds for Scottish salmon on the consumer and stakeholders’ side. A general consensus was found among consumers, with only 10% of the sample opposed to the inclusion of insects into fish feed, and stakeholders, provided the feeds are proven to be safe, reliable, and cost effective. To the best of our knowledge, only two recent papers have focused on determinants of consumers’ acceptance of insects in animal feed via experimental procedures [56,57]. Ankamah-Yeaboah et al. [56] analyzed data collected from an online questionnaire administered to 610 German consumers, using a discrete choice experiment (DCE) in order to resemble a real market decision-making setting. According to their results, most consumers interviewed were not concerned about the type of feed. In contrast, 23% of the sample showed negative opinions towards fish reared with insects but a positive preference towards convenience. Therefore, the authors argued that “to maintain market share in this segment, producers using insect-based feed may want to develop fish products that are perceived as relatively convenient to prepare”. Bazoche et al. [57] identified three hypotheses assessing consumers’ willingness to choose products fed with insects; characterizing the relationships between consumers’ acceptance of insects as feed and food neophobia, disgust, and personality traits; and evaluating the role of positive information upon consumers’ willingness to choose products from animals fed with insects. The results seemed to show that information about the environmental impact of feeding methods in aquaculture may influence consumer choice. The preference for animal feeding with insect meal could be improved with information campaigns on the negative impact of traditional feeding practices.

### 3.2. Sensory Studies

The first studies that tried to evaluate consumer acceptability of animal products fed with insects (i.e., soldier fly larvae) using a taste evaluation test were conducted many decades ago [60,61,62].

Not all the studies investigating the dietary effects of insect meal on performance included a sensory and consumer test to evaluate the potential changes with respect to meat quality. However, those which included a sensory analysis showed that there are no major effects on aroma, texture, or off-flavors in the meat quality of species fed with traditional feeds compared with those fed with an insect meal such as HI prepupae [3,61,63,64,65] or TM larvae [66].

For instance, Sealey et al. [25], using a triangle difference test with 30 untrained panelists, found no discernible sensory attribute differences between fish fed with HI prepupae and those fed with a fish meal control diet.

Even if no off-flavor was reported, Borgogno et al. [63] found a dominance of metallic flavor in fillets of fish fed HI diets that could be perceived as unfamiliar to the consumer, while Belghit et al. [64] reported no significant relationship between the dietary inclusion level of HI and any of the sensory properties evaluated.

Khan et al. [66] carried out a study to compare organoleptic characteristics of broiler chicken meat fed with different kinds of insect meal (i.e., maggot meal, silkworm meal, and mealworm) and found no effects on the sensory profile.

A study by Sun et al. [67] found significantly higher scores for chewiness, flavor, aroma, and overall appreciation for the breast and thigh meat of male broilers, which were reared on grassland containing a large population of grasshoppers rather than a control sample reared on a maize–soybean diet. Instead, no significant differences were discerned with respect to color and juiciness between the two treatments. However, in their study, as the authors stated, it was difficult to understand whether the superior sensory attributes might be due to the grasshopper feed or the wider dietary choice and increased locomotion offered in a free-range environment.

We found trends regarding the limitations of this body of work. Some authors [1,63,68,69,70] specified crucial information on how the sensory trial was conducted; they included information such as how many judges were involved in the sensory test, how the flesh was prepared (type and size of cut) and cooked (e.g., fried, boiled), and what kind of hedonic scales were used for rating the panel’s preferences. However, other articles did not include specific information on the taste panel; they omitted information such as whether the panelists were trained or not, whether any information about the sample was supplied to participants, whether a common attribute vocabulary for profiling was developed, how the acceptability questions were framed, and whether it was a blinded test. This lack of details impedes our understanding of the reliability of the sensory findings.

In conclusion, from the studies described so far, the replacement of fish/soy meal components in the diet of some farmed fish and poultry species do not present negative effects on the flavor, juiciness, or texture of the final products.

However, the potential effect of insect feed on meat quality traits and sensory properties should be further investigated in order to understand consumer acceptance and develop marketing strategies [71].

### 3.3. Stakeholders’ Perspective

Stakeholders’ perspectives on the topic of insects as feed have been investigated a few times and mainly in African countries [72,73], where insects are commonly used as a feed source (see Kenis et al. for a review [74]). Ssepuuya and colleagues [73] investigated stakeholders’ perceptions of the use of insects as feed through a cross-sectional survey. Their findings show that, at least in Uganda, the majority of stakeholders are aware of this alternative use of insects, thanks to their own experience, and have a positive attitude towards the subject. Despite this high rate of awareness, only a small percentage ever reared or used insects for feeding fish.

In the European studies, the first attempt to survey stakeholders and farmers was conducted by Verbeke et al. [52]. Considering their perspective, farmers were more critical of the use of insects in animal feed than agriculture sector stakeholders or citizens; farmers consistently had weaker perceptions of benefits and stronger perceptions of the risks associated with the use of insects in animal feed. Furthermore, despite a general positive attitude and claimed acceptance, only 25% of livestock farmers indicated an intention to use insects as feed; risks, future consumer acceptance, communication, and attitude of retailers were among the major concerns outlined by respondents.

In a qualitative study, Marberg et al. [28], conducted 19 in-depth, semi-structured interviews with experts and stakeholders, including breeders, industry experts, researchers, government officials, and livestock farmers, in the emerging sector. Their results highlighted many actions that could improve the “insects as feed” sector and its acceptance among consumers: (a) spreading knowledge regarding the subject (e.g., communicating potential benefits through the involvement of stakeholders in order to improve consumer trust [75,76]) and how potential risks are mitigated [77,78]; (b) prioritizing transparency to avoid the “social amplification problem” [75]; and (c) demonstrating a sense of urgency to overcome consumers’ limited support for new food technologies when urgency is not felt [76].

From a marketing point of view, it is interesting to observe how some insect production companies (e.g., Protix, https://protix.eu/products_by_protix/#oerei) are promoting insects as a feed related to a “natural diet” for chickens and good for their “natural behavior”. In the future, it would be of interest to understand how the private sector will develop its advertising campaigns for animal farmers and whether/how consumers will receive this type of information.

## 4. Future Perspectives of the Feed Farming Industry

According to the International Platform of Insects for Food and Feed (IPIFF), the insect protein industry will have to face three main challenges in order to reach its full potential.

First, the insect industry will need to considerably scale up [22]. Indeed, the current trading price of insect meals is not yet competitive enough. Additionally, production volumes of fishmeal, high-quality soybean meal extract, and soybean meal is hundreds or thousands of times larger than protein products obtained from insects [15]. Therefore, by increasing the scale of production, insect farmers will be able to increase the price competitiveness and stability of their products compared with other sources of protein. In particular, automation and controlled production systems will significantly help stakeholders to achieve this goal by making insect production less labor-intensive [22].

Secondly, as mentioned before, livestock farmers in the EU must regularly meet consumers’ expectations for safe, nutritious, and high-quality products of animal origin. Furthermore, they are expected to address societal challenges, such as reducing the use of antibiotics, in order to face antibiotics-resistance issues. As a consequence of this trend, insect producers will need to produce nutritious and high-quality products in order to respond to these new demands [22].

The final challenge is represented by the General Food Law, whose principles will have to be strictly followed by insect producers. In EU countries, opportunities for using and feeding insects are still limited due to legislative issues [24]. Insects are currently not allowed to be used as feed for poultry and pigs (even if the amendments pass as expected in 2020) and may not be fed with former foodstuff containing meat, fish, or food losses originating from restaurants or catering establishments [22].

In the future, two main research topics will need to be further investigated. In order to establish the economic impact of introducing insects into animal feeding and more cost–benefit analysis will have to be regularly carried out to deeply investigate how these alternative ingredients effectively influence overall production costs. In particular, the offset of the extra costs of novel feeds by the improvement of animal health and performance, as well as the market premium potentially derived from higher welfare products, will have to be taken into account. As a second line of research, more attention will have to be focused on the use of insects as feed additives to modulate and improve the gut health of production animals. In particular, multidisciplinary techniques (such as histomorphology, histochemistry, immunohistochemistry, molecular biology, and NGS) will be strongly recommended to characterize the gut health status of insect-fed animals.

The few consumer studies carried out so far suggest that acceptance will not be a barrier towards the development of the insect protein industry for feed. However, it will be of interest to understand whether the use of a more sustainable feed source might increase the willingness to pay for animal products fed with insects and whether the overall acceptability, also from a sensory point of view, will be perceived better than conventional products.

## 5. Conclusions

As mentioned above, edible insects remain underutilized in the animal feed industry; however, with the rapid development of intensive industrial insect farming, their potential use is expected to increase [71,79,80].

Most of the trial studies, which aim to investigate the contribution of insects as a feed source in animal diets, mainly focus on growth performance, microbiological and health implications, and nutrient composition and utilization, whereas the consumer’s response has never been studied with a rigorous approach. Common techniques and methods familiar among consumers and sensory scientists have not been used so far.

Future studies on insects as feed should better investigate the consumer’s point of view about this alternative protein source in farm animals. Among the main issues on which to focus are the following: evaluating effects on sensory perception, measuring potential consumers’ willingness to pay and preferences for this new attribute, assessing the role of the neophobia factor and other relevant individual traits of consumers, and identifying risks and benefits associated with the introduction of this novel feed. Potential differences in the main insect species used and animals involved should be taken into consideration for marketing and communication strategies.

## Figures and Tables

**Table 1:** Feed legislation on the use of insects as feed.

Country	Authority	Regulation	Insects as Feed
European Union (EU)	EFSA	EU Decisions/regulations	PAPs authorized in aquaculture Authorized fat from insects in feed Positive list of rearing insects
USA	FDA	FFDCA	Additive approval list or GRAS needed for insects. HI larvae included as ingredient for animal food
Canada	CFIA	FAFR	Feed raw material needs authorization, HI product authorized for poultry.
North Korea	Ministry of Agriculture, Food and Rural Affairs	Not present	Prohibited
South Korea	Ministry of Agriculture, Food and Rural Affairs	Not present	Does not required authorization
China	none	Not present	Does not required authorization

EFSA: European Food Safety Authority; PAPs: Processed Animal Proteins; FDA: Federal Food and Drug Administration; FFDCA: Federal Food, Drug, and Cosmetic Act; GRAS: Generally Recognized as Safe; HI: *Hermetia illucens*; CFIA: Canadian Food Inspection Agency; FAFR: Food Act and Feeds Regulation.

**Table 2:** Overview of surveys on consumer acceptance of insects as feed.

Study	Country	Method	Sample	Product	Main Findings
Ankamah-Yeboah et al., 2018 [56]	Germany	Discrete Choice Experiment	610 consumers	Fish (rainbow trout)	23% of the sample exhibit negative preferences towards insects as feed in trout production. Consumption would rise if price were reduced or other attributes, such as convenience aspects, were improved.
Bazoche and Poiret, 2016 [57]	France	Hypothetical Choice Experiment	327 consumers (selected from a consumer panel)	Fish (smoked trout fillets)	Information about the environmental impact of feeding methods in aquaculture may influence consumer choice.
Kostecka et al., 2017 [58]	Poland	Survey	210 consumers	Beef, pork, poultry, fish	Positive attitudes about using insects to feed cattle and pigs were expressed by 41.8% and 47.2% of the sample. Slightly higher approval was expressed by the respondents for meat from birds (chicken 58.1%) and fish (56.7%) fed in a similar way.
Laureati et al., 2016 [54]	Italy	Survey	341 participants contacted via the web/social networks, of which 68 also performed a visual hedonic assessment	Fish and livestock	53% of the consumers declared themselves to be ready to incorporate insects into animal diets and to eat fish and livestock reared with insect-containing feed.
Mancuso et al., 2016 [55]	Italy	Survey	277 Northern Italian consumers	Fish	Almost 90% of consumers have a positive attitude towards using insect meal as feed, and most of the respondents intend to purchase and eat farmed fish, even those fed with insect meals, so long as hygiene requirements are met. Interest is mainly affected by socioeconomic variables, knowledge of the issue, and interest attributed to origin and certification. Positive attitude is mainly influenced by interest in this issue and variables linked to appearance and price, whereas the willingness to buy fish fed with insect meals is closely linked to the importance of price.
Popoff et al., 2017 [59]	United Kingdom (UK)	Semi-structured Interviews Survey	4 industry stakeholders 200 consumers	Fish (Scottish Atlantic salmon)	Salmon producers would not be opposed to the use of insect materials, provided they were traceable, safe, cost-competitive, and did not impact the quality of their products. Most consumers would be willing to accept the use of insects as feed for salmon. Taste was rated a very important indicator for purchasing decisions.
PROteINSECT, 2016 [52]	Worldwide	Two surveys on consumer perception	Study 1 in 2014: 1302 responses Study 2 in 2015: 1150 responses		Study 1: 88% said that more information on the use of insects as a feed source should be made available. 57% of respondents thought that there should be appropriate labeling of fish, chicken, or pork fed on insect protein. Study 2: 70% of respondents considered it acceptable to feed insect protein to farmed animals including fish. 66% of respondents would be very comfortable eating meat from a farmed animal fed on insect meal. 64% of respondents said there is no risk or low risk to human health in eating farmed animals fed on insect meal.
Verbeke et al., 2015 [53]	Belgium	Survey	415 participants: 87 citizens 137 stakeholders 196 farmers	Fish, poultry, pigs	Attitudes and acceptance of farmers, agriculture sector industry stakeholders, and citizens towards the use of insects in animal feed are generally favorable, especially for fish and poultry. Insect-based feed was perceived to be more sustainable with better nutritional value but a lower microbiological safety. Foods obtained from animals fed on insect-based feed were perceived to be more sustainable, to have a better nutritional value, and to be healthier. By contrast, the resulting foods were associated with possible off-flavors and the presence of allergens.
